# Research progress on digital-twin-driven full life-cycle design and manufacturing of rehabilitation assistive devices

**DOI:** 10.3389/fbioe.2026.1820883

**Published:** 2026-09-15

**Authors:** Hang Tian, Xueyan Song, Dan Li

**Affiliations:** 1 Department of Design, Dalian Art College, Dlian, China; 2 Department of Mechanical Engineering, Dalian University, Dlian, China

**Keywords:** clinical translation, digital twin, human– device interaction, lifecycle framework, rehabilitation assistive devices

## Abstract

Digital Twin (DT) technology has emerged as a promising paradigm for the design, optimization, and lifecycle management of rehabilitation assistive devices. However, existing studies are often limited to specific technologies or applications, lacking a systematic perspective on different device categories and their lifecycle requirements. This review summarizes recent advances in DT-driven rehabilitation assistive devices. A DT-oriented taxonomy is proposed, classifying rehabilitation assistive devices into structure-oriented, human–device coupled, and intelligent interactive devices according to their functional requirements. Based on this taxonomy, a lifecycle-oriented DT framework is established, encompassing multimodal data acquisition, digital modeling, simulation and optimization, manufacturing integration, and closed-loop feedback updating. Key enabling technologies, including biomechanical modeling, finite element analysis, artificial intelligence, multi-source data fusion, and additive manufacturing, are systematically reviewed. Representative applications are further analyzed to illustrate the role of DT in personalized design, performance prediction, adaptive control, and intelligent manufacturing. The review indicates that DT technology can significantly improve personalization, performance optimization, and lifecycle management of rehabilitation assistive devices. Despite challenges in data interoperability, real-time computation, and clinical validation, DT is expected to play an increasingly important role in advancing intelligent, precise, and personalized rehabilitation. This review provides a structured reference for future research and practical implementation of DT technologies in rehabilitation engineering.

## 1 Introduction

Rehabilitation assistive devices play a critical role in restoring functional mobility, enhancing independence, and improving quality of life for individuals affected by neurological disorders, musculoskeletal impairments, traumatic injuries, and age-related functional decline. Common rehabilitation assistive devices include orthotic devices, prosthetic systems, powered exoskeletons, and wearable rehabilitation platforms, each serving distinct biomechanical and therapeutic functions (Shi et al., 2019). With the rapid growth of the aging population and the increasing prevalence of chronic disabilities worldwide, the demand for personalized rehabilitation interventions has risen substantially. However, the development of conventional rehabilitation devices still largely relies on empirical design approaches, iterative physical prototyping, and clinical experience, resulting in prolonged development cycles, high manufacturing costs, and limited adaptability to individual patient needs (Nouri et al., 2023). Moreover, rehabilitation outcomes are strongly influenced by patient-specific anatomical characteristics, biomechanical properties, disease progression, and rehabilitation behaviors. Consequently, traditional one-size-fits-all design strategies often fail to meet the growing demand for personalized rehabilitation solutions.

Digital Twin technology has emerged as a transformative paradigm for addressing the limitations of conventional rehabilitation-device development. Broadly defined, a digital twin is a virtual representation of a physical entity that continuously exchanges information with its physical counterpart through bidirectional data communication, enabling real-time monitoring, simulation, prediction, optimization, and decision support throughout the system lifecycle. Originally developed for aerospace and intelligent manufacturing applications, DT technology has rapidly expanded into healthcare and biomedical engineering, driven by advances in medical imaging, wearable sensing, artificial intelligence, cloud computing, and additive manufacturing technologies (Fukawa and Rindfleisch, 2023).

Within the field of rehabilitation engineering, DT technology offers new opportunities for establishing personalized virtual models of patients, assistive devices, and rehabilitation environments. By integrating multimodal physiological data (Farhan et al., 2021), biomechanical models (Hussain et al., 2022), intelligent optimization algorithms (Lin et al., 2020), and manufacturing systems (Shahar et al., 2020), DT technology can support personalized design, virtual testing, adaptive control (Lugrís et al., 2024), and lifecycle management of rehabilitation assistive devices (Zell and Rosenhahn, 2020).

Despite the rapid growth of DT-related research in rehabilitation engineering, several important challenges remain unresolved. First, most existing studies focus on isolated stages of the rehabilitation-device lifecycle, such as anatomical reconstruction, biomechanical simulation, intelligent control, or additive manufacturing. Limited attention has been devoted to integrating these components into a unified DT-enabled lifecycle framework. Second, rehabilitation assistive devices constitute a highly heterogeneous technological domain. Orthotic devices primarily emphasize structural optimization and anatomical fitting; prosthetic systems require close human–device biomechanical integration; powered exoskeletons rely heavily on real-time control and adaptive algorithms; whereas wearable rehabilitation systems focus on continuous monitoring and rehabilitation assessment. Nevertheless, relatively few studies have investigated how DT architectures should be adapted to accommodate the varying requirements of different rehabilitation-device categories. Third, despite significant technological advances, critical challenges related to data interoperability, real-time model updating, clinical validation, user-centered design, and regulatory translation remain insufficiently addressed. Finally, existing review articles have predominantly focused on specific technologies or application scenarios. A comprehensive review that systematically integrates rehabilitation-device taxonomy, DT architecture, simulation and optimization, manufacturing integration, clinical validation, and lifecycle feedback remains lacking.

To address these gaps, this review presents a comprehensive and critical analysis of DT technologies for rehabilitation assistive devices.

The main contributions of this review are fourfold:

1. A taxonomy of rehabilitation assistive devices is established, encompassing structure-oriented devices, human–device coupled devices, and intelligent interactive devices, thereby clarifying their distinct DT requirements.
2. A lifecycle-oriented DT framework is proposed, covering data acquisition, digital modeling, simulation and optimization, manufacturing integration, and closed-loop feedback.
3. Representative implementation pathways and enabling technologies are comparatively analyzed across different categories of rehabilitation assistive devices.
4. Current technological limitations, clinical validation challenges, regulatory considerations, and future research opportunities are critically discussed from both engineering and clinical perspectives.

## 2 Taxonomy of rehabilitation assistive devices from a digital twin perspective

### 2.1 Device classification from a digital twin perspective

Traditional classification schemes for rehabilitation assistive devices are typically based on anatomical location (Luo et al., 2024), clinical function (Pei et al., 2026), or device morphology, such as upper (Tian et al., 2025) and lower-limb devices (Scherb et al., 2023), orthotic (Lin et al., 2024) and prosthetic systems (Guo et al., 2025), or passive (Aykora et al., 2025) and active rehabilitation technologies (Berger and d’Avella, 2024). Although these classifications are valuable in clinical practice, they do not adequately reflect the diverse implementation requirements of digital twin systems.

From a DT perspective, the complexity of a rehabilitation device is primarily determined by its requirements for virtual modeling, data acquisition, model updating, control interaction, and lifecycle optimization. Consequently, devices with different clinical functions may employ similar DT architectures, whereas devices belonging to the same clinical category may require fundamentally different DT implementation strategies.

To better characterize these differences, this review establishes a DT-oriented taxonomy based on the core functional requirements of DT technologies in rehabilitation assistive devices. Accordingly, rehabilitation assistive devices are categorized into three groups: (1) structure-oriented devices, (2) human–device coupled devices, and (3) intelligent interactive devices. This taxonomy provides a theoretical basis for analyzing how DT architectures should be adapted to different categories of rehabilitation devices.

### 2.2 Structure-oriented rehabilitation devices

Structure-oriented rehabilitation devices primarily rely on mechanical structures to provide support, correction, stabilization, or load redistribution (Lu et al., 2025). Typical examples include ankle–foot orthoses (AFOs), knee–ankle–foot orthoses (KAFOs), spinal orthoses, and passive prosthetic components. Figure 1.

**FIGURE 1:**
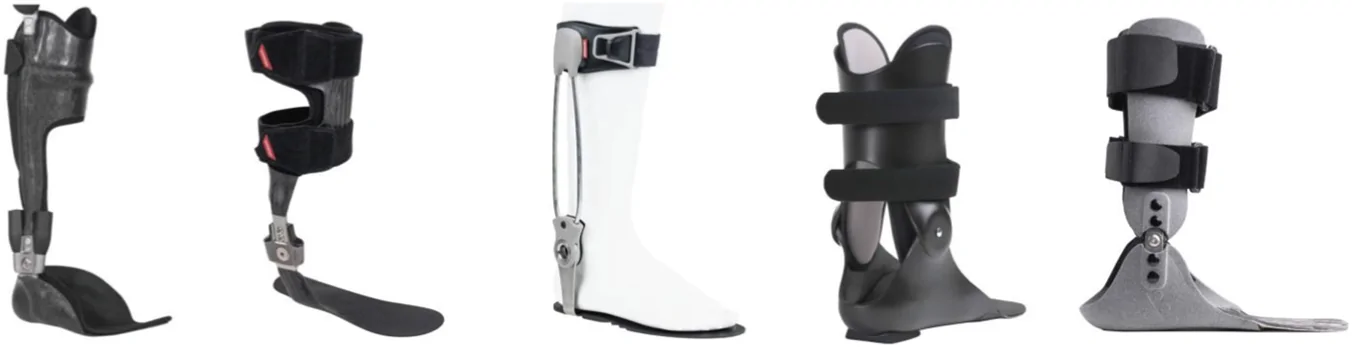
Schematic diagram of structure-oriented rehabilitation equipment.

For these applications, the primary objective of DT implementation is to establish accurate geometric and biomechanical models of the human–device system. Patient-specific anatomical reconstructions derived from medical imaging, finite element modeling, and structural optimization constitute the foundation of the DT model.

Because these devices generally exhibit stable mechanical behavior and limited active control functionality, their requirements for real-time synchronization are relatively modest. Consequently, DT technologies are primarily employed for personalized design, structural performance prediction, comfort assessment, and additive-manufacturing-enabled customization. Therefore, the key DT requirements for structure-oriented devices include geometric fidelity, biomechanical simulation accuracy, and manufacturing integration.

### 2.3 Human–device coupled rehabilitation devices

Human–device coupled rehabilitation devices are characterized by strong biomechanical interactions between the user and the assistive device, resulting in continuous virtual–physical mapping and dynamic system evolution (Uhlenberg et al., 2023). Representative examples include dynamic prosthetic systems, microprocessor-controlled prostheses, and intelligent orthotic devices with adaptive adjustment capabilities. Figure 2.

**FIGURE 2:**
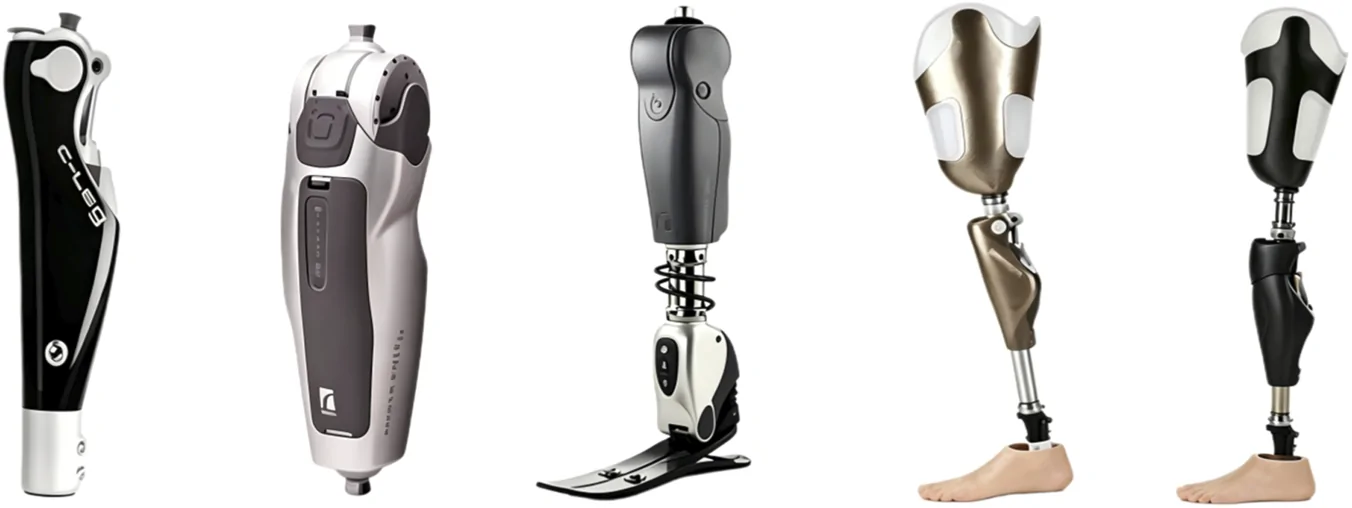
Schematic diagram of human-machine collaborative rehabilitation equipment.

Compared with structure-oriented devices, these systems require DT technologies not only to capture structural characteristics but also to model the dynamic interactions between human biomechanics and device behavior. As a result, musculoskeletal modeling, gait analysis, interface pressure prediction, and movement adaptation become essential components of the DT system (Zhang et al., 2024).

Furthermore, wearable sensing technologies—including inertial measurement units (IMUs), electromyography (EMG), and pressure sensors—are commonly integrated to support model updating and performance evaluation (Yang et al., 2024). Consequently, DT systems for this category place greater emphasis on biomechanical coupling, adaptability, and long-term assessment of user status.

### 2.4 Intelligent interactive rehabilitation devices

From a DT perspective, intelligent interactive rehabilitation devices represent the most complex category of rehabilitation technologies. Typical examples include powered exoskeletons, rehabilitation robots, and intelligent assistive systems capable of autonomous adaptation and decision-making. Figure 3.

**FIGURE 3:**
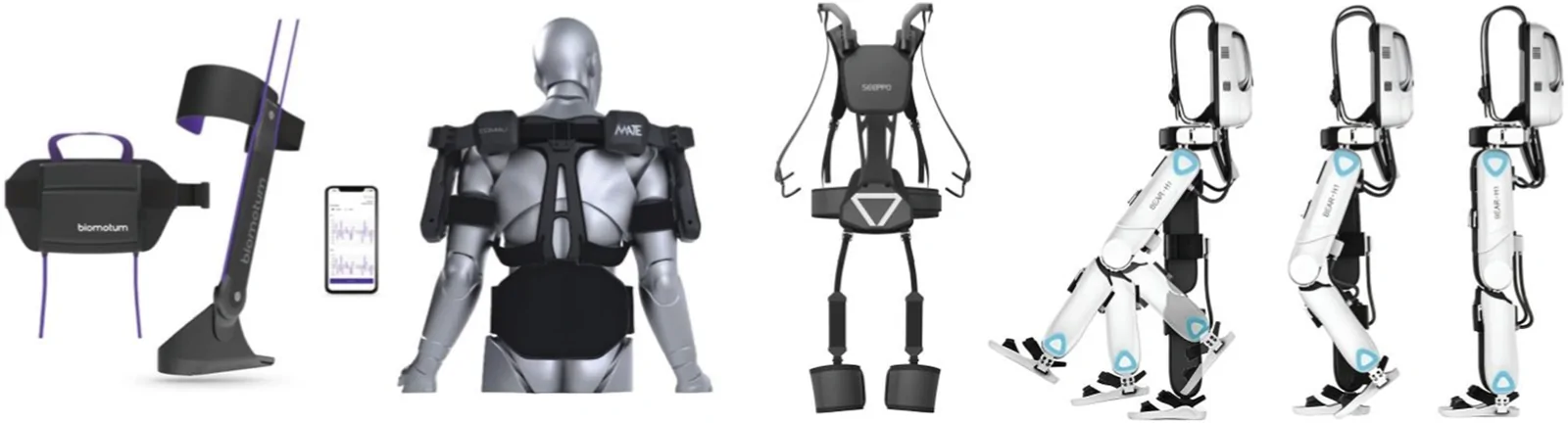
Schematic diagram of intelligent interactive rehabilitation equipment.

These devices rely heavily on real-time sensing, coordinated actuator control, adaptive algorithms, and human–machine interaction (Bogaert et al., 2026). Accordingly, their DT systems must continuously synchronize virtual and physical states while simultaneously supporting prediction, optimization, and control functions (Fırat, 2026).

Unlike the previous categories, DT systems for intelligent interactive devices typically integrate artificial intelligence, reinforcement learning, cloud computing, and edge computing technologies. Their objective extends beyond representing the physical system to enabling adaptive decision-making and predictive intervention. Consequently, intelligent interactive rehabilitation devices impose the highest requirements on data acquisition, computational efficiency, real-time communication, and closed-loop control.

### 2.5 Chapter summary

The proposed taxonomy highlights the substantial differences among rehabilitation assistive devices in terms of functional objectives, human–device interaction mechanisms, and technological complexity. As a result, their DT implementation strategies differ significantly with respect to modeling approaches, data requirements, real-time synchronization demands, and lifecycle management objectives.

As summarized in Table 1, structure-oriented rehabilitation devices primarily focus on anatomical reconstruction, structural simulation, and manufacturing-driven customization. Their DT models emphasize geometric accuracy, finite element analysis, and personalized manufacturing. Human–device coupled rehabilitation devices additionally require biomechanical adaptability and interface interaction modeling, leading to a greater reliance on wearable sensing technologies, musculoskeletal modeling, and dynamic performance assessment. In contrast, intelligent interactive rehabilitation devices demand continuous synchronization between physical and virtual systems and place the highest requirements on real-time communication, adaptive control, and predictive decision-making.

**TABLE 1:** Digital-twin-oriented taxonomy and functional requirements of rehabilitation assistive devices.

Category	Representative devices	Primary DT objective	Data requirement	Real-time requirement	Manufacturing integration
Structure-oriented	AFO, KAFO, passive prosthesis…	Structural optimization	Geometry, mechanics	Low	High
Human-device coupled	Prosthesis, smart orthosis…	Biomechanical adaptation	Gait, Interaction	Medium	Medium
Intelligent interactive	Exoskeleton, rehabilitation robot…	Control and prediction	Multimodal sensorData	High	Medium

Despite these differences, a common conclusion emerges: all categories rely on a shared DT workflow consisting of data acquisition, virtual modeling, performance evaluation, optimization, and feedback-driven updating. The distinction lies not in the existence of these processes, but in their relative importance, implementation strategies, and computational requirements.

Therefore, DT technologies for rehabilitation assistive devices should not be regarded as isolated solutions tailored to specific products. Instead, they should be viewed as customized implementations of a common lifecycle-oriented DT paradigm. This perspective provides the theoretical foundation for establishing a unified DT framework that supports diverse rehabilitation-device applications while retaining sufficient flexibility to accommodate their unique functional requirements.

Based on these observations, the next chapter proposes a lifecycle-oriented DT framework for rehabilitation assistive devices. The framework integrates multimodal data acquisition, digital modeling, simulation and optimization, manufacturing integration, and closed-loop feedback within a unified architecture, while emphasizing adaptive implementation pathways for different categories of rehabilitation devices.

## 3 Key applications of digital twin technologies in different categories of rehabilitation assistive devices

Building upon the taxonomy proposed in Chapter 2, a lifecycle-oriented digital twin framework is established to support the design, optimization, manufacturing, deployment, and continuous evolution of rehabilitation assistive devices. Unlike conventional digital modeling approaches that focus on isolated stages of product development, this framework emphasizes bidirectional information exchange and continuous synchronization between physical and virtual systems throughout the entire device lifecycle.

Within this framework, DT technologies play distinct roles across different categories of rehabilitation assistive devices. The key implementation components include multimodal data acquisition and fusion, parametric and biomechanical modeling, real-time virtual–physical mapping and model updating, performance prediction, and algorithm-driven closed-loop optimization. Together, these processes constitute the core architecture of DT systems and provide the foundation for geometric fidelity, biomechanical accuracy, and subsequent manufacturing integration. Figure 4.

**FIGURE 4:**
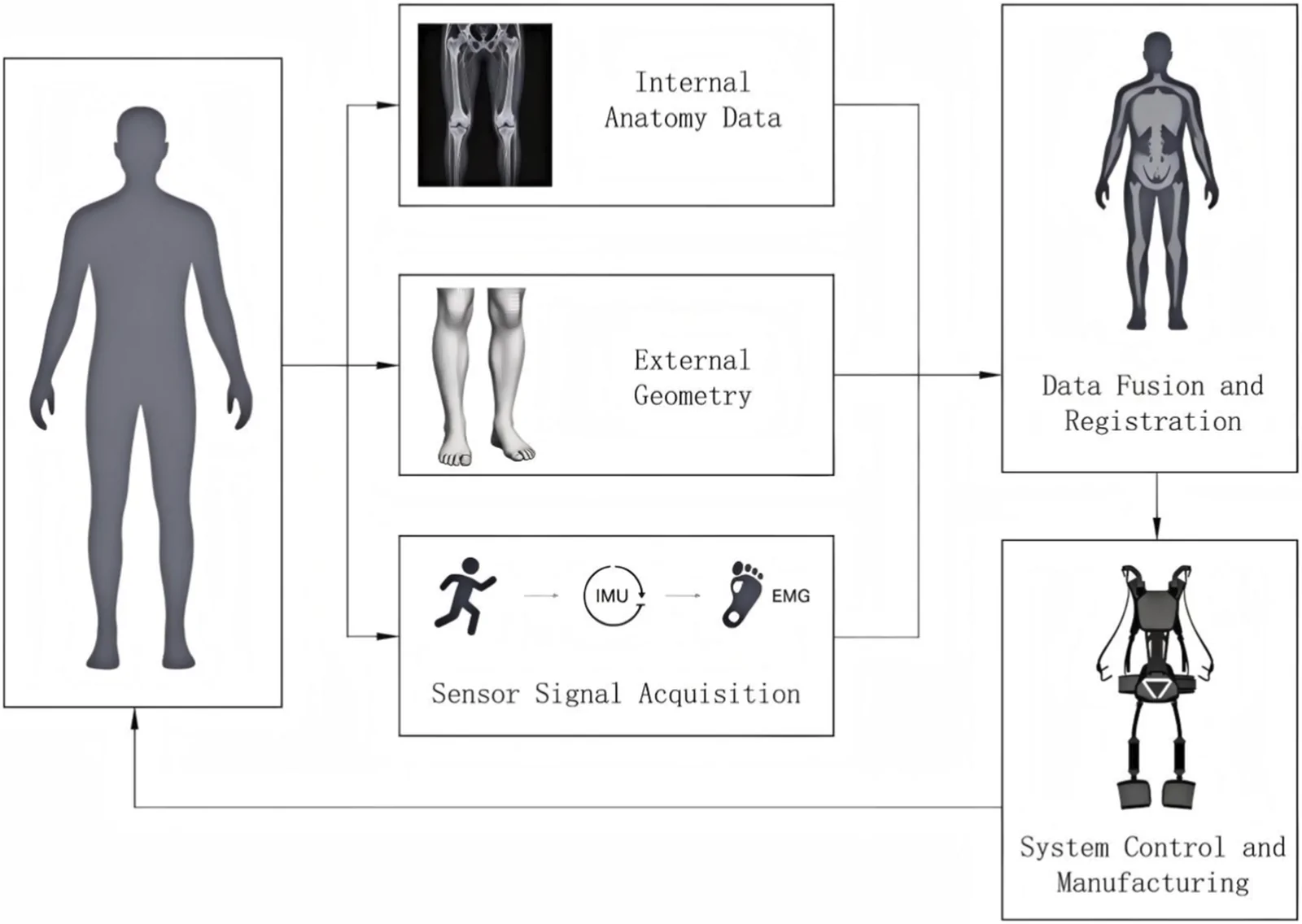
Digital twin modeling workflow for rehabilitaion assistive devices.

### 3.1 Multimodal data acquisition and synchronization

The accuracy and reliability of rehabilitation-device digital twins fundamentally depend on the quality, diversity, and temporal consistency of data acquired from physical systems. In rehabilitation engineering, data sources are inherently heterogeneous and may include medical imaging (Abhisheka et al., 2023), biomechanical measurements (Werling et al., 2023), wearable sensor data (Khan et al., 2024), embedded device sensors (Bonfiglio et al., 2024), manufacturing records (Dorschky et al., 2025), and clinical outcome assessments (De Pasquale et al., 2025).

To establish accurate virtual representations, multimodal data must be integrated through standardized procedures for data acquisition, preprocessing, synchronization, and management. Common data sources include: internal anatomical information (CT, MRI); external geometric morphology (structured-light or laser point-cloud scanning); and dynamic functional data (motion capture systems, inertial measurement units [IMU], plantar-pressure sensors, and electromyography [EMG]). CT and MRI provide high-resolution volumetric information on bones, soft tissues, and joints, enabling precise extraction of bone geometry, joint spacing, and tissue volumes (Abhisheka et al., 2023). Structured-light or laser scanning rapidly captures surface curvature and local fitting regions, supporting external-shell design and comfort optimization (Silva et al., 2024). Gait capture and IMUs record joint angle trajectories, step frequency, and acceleration; EMG reveals muscle activation timing; plantar-pressure distribution reflects contact mechanics during motion (Werling et al., 2023).

To integrate these heterogeneous data sources, researchers employ algorithms such as point-cloud registration, surface reconstruction, and voxel interpolation to align geometric and physiological datasets within a unified coordinate system and ensure topological consistency. Filtering, feature matching, and error-correction strategies are widely used to eliminate systemic noise and geometric deviations. In addition, deep learning has been incorporated into segmentation and feature-extraction processes to automatically identify bone boundaries, muscle contours, and surface morphology, significantly improving modeling speed and robustness while maintaining high accuracy (Suganyadevi et al., 2022).

### 3.2 Digital modeling and virtual representation

The defining characteristic of human–device coupled rehabilitation assistive devices is the continuous virtual–physical mapping and dynamic evolution between the physical system and its digital counterpart (Li et al., 2024). Through data-driven mechanisms, digital twin technology enables real-time synchronization and adaptive updating of the virtual model, allowing it to accurately reflect the state, behavior, and performance of the physical rehabilitation device throughout its operational lifecycle (Fırat, 2026). In this process, sensor, model, and control layers form a high-frequency closed-loop system, allowing the virtual twin to faithfully reflect the actual structural state and functional behavior of the physical device. This enables dynamic performance evaluation and intelligent control of rehabilitation assistive technologies.

At the sensing layer, high-fidelity acquisition and synchronized transmission of sensor data form the necessary foundation. Rehabilitation devices commonly integrate multiple types of sensors to measure human motion states, joint torques, contact pressures, and muscle activity signals (Rana and Mittal, 2021). Data are transmitted in real time via wireless modules to cloud or edge computing platforms, where they are matched and fused with corresponding variables in the virtual twin (De Koning et al., 2023). Through time-synchronization and spatial-registration algorithms, the system achieves multichannel data-driven updates under varying sampling rates and transmission delays (Bariah and Debbah, 2023).

At the model layer, the virtual twin undergoes dynamic updating via data assimilation and model-correction mechanisms. Ward (Ward et al., 2021) utilized particle filtering to continuously calibrate physical models, enabling efficient estimation and real-time updating of time-varying parameters, thereby significantly improving environmental responsiveness and prediction accuracy. Soykan (Soykan et al., 2025) addressed mismatches between discrete-event simulation and real-time data streams by developing a time-window-smoothed particle filtering algorithm that bridges event-driven and fixed-interval measurements, resulting in more reliable state synchronization and real-time data assimilation for digital twins.

At the control layer, the coupled virtual–physical model supports adaptive control and intelligent optimization of rehabilitation devices. For example, He (He et al., 2023) integrated master–slave design, event state machines, and control services into a digital-twin framework that incorporates carbon-footprint analysis, enabling environmentally informed adaptive control and optimization for physical systems. Franciosa (Franciosa et al., 2020) developed a digital-twin framework combining sensor data, deep learning, and multiphysics CAE simulation for product assembly systems, achieving root-cause diagnosis, self-correction, and real-time optimization of quality defects—significantly improving final product yield. This closed-loop digital-twin control mechanism illustrated in Figure 5 establishes a dynamic coupling from “perception–modeling–control–feedback,” enabling intelligent adaptation and predictive optimization of rehabilitation devices.

**FIGURE 5:**
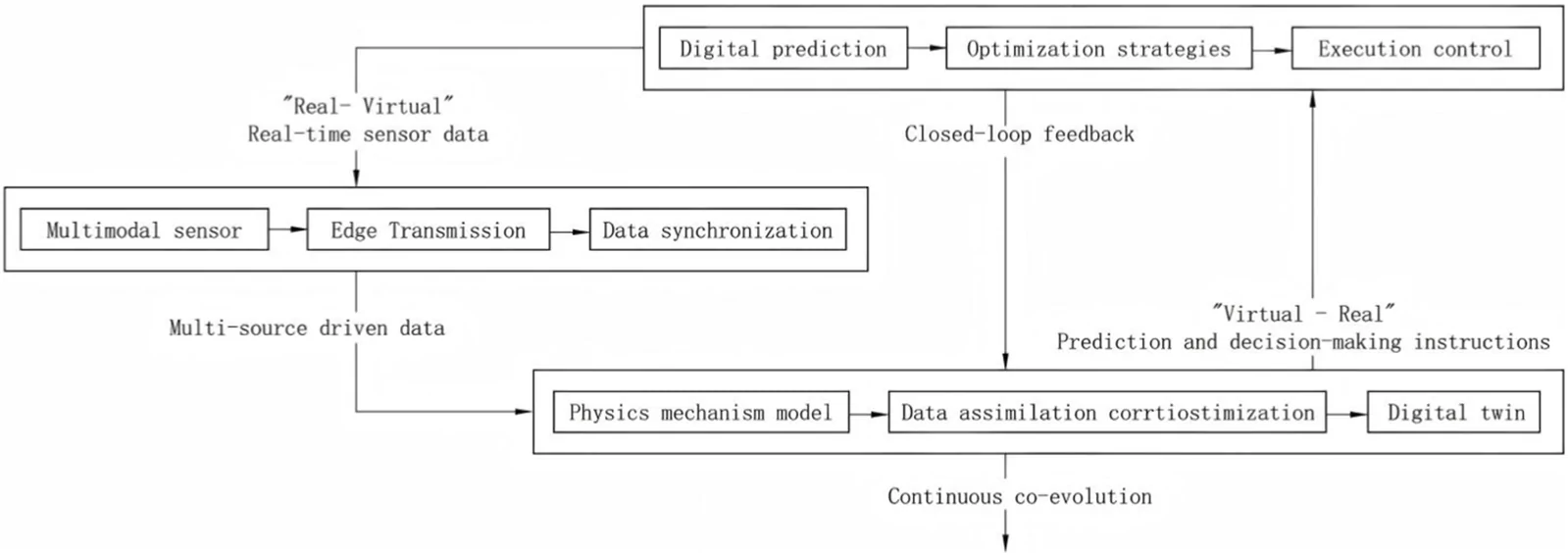
Digital twin mapping framework.

### 3.3 Performance optimization and intelligent interaction

For intelligent interactive rehabilitation devices, once multimodal data fusion and virtual–physical mapping mechanisms have been established, the digital twin model is capable of dynamically and accurately representing both patient-specific physiological characteristics and device responses (Liao et al., 2025). Building upon this foundation, digital twin technologies can be further utilized for performance simulation, design exploration, and intelligent optimization, enabling the transition from model representation to decision-driven operation (Liu et al., 2024).

By replicating real-world usage scenarios within a virtual environment and conducting parametric analyses, the system can proactively identify potential risks, balance multiple design objectives, and generate personalized optimization strategies. This capability allows digital twins not only to predict system performance under varying conditions but also to support adaptive decision-making throughout the rehabilitation process.

In terms of performance optimization, Esrafilian et al. (Esrafilian et al., 2022) employed finite element analysis (FEA) by integrating a fiber-reinforced viscoelastic soft-tissue model with a skeletal finite element model to quantify stress and strain distributions within knee joint tissues at different rehabilitation stages. Their results revealed substantial inter-individual biomechanical variability during rehabilitation exercises, providing a simulation-based foundation for patient-specific rehabilitation planning and tissue-load optimization.

Mokadim et al. (Mokadim et al., 2024) proposed a simulation-driven design methodology for personalized AFOs. By combining OpenSim-based multibody dynamic simulations with MATLAB/Simulink control modeling, they developed a hybrid actuation mechanism capable of decomposing ankle quasi-stiffness profiles. Compared with unassisted walking, the proposed design reduced peak ankle joint torque by approximately 19.24% and decreased overall muscle activity by 17.43% during load-bearing gait tasks, demonstrating the potential of simulation-based optimization for individualized orthotic design.

Similarly, Mohammadi et al. (Mohammadi et al., 2024) developed a lightweight ankle–foot orthosis powered by dielectric elastomer artificial muscles. Through theoretical and biomechanical analyses, the device was shown to provide up to 69% of the required plantarflexion assistive torque and 100% of the dorsiflexion assistance throughout the gait cycle. These findings highlight the importance of simulation-based evaluation for predicting assistive performance and optimizing rehabilitation-device design.

The key characteristics of the methodologies adopted in these representative studies are summarized in Table 2. Despite differences in modeling approaches and device architectures, all three strategies demonstrate the critical role of simulation, prediction, and optimization in supporting the development of intelligent interactive rehabilitation devices.

**TABLE 2:** Core simulation strategies in digital twin.

Simulation strategy	Core principle	Scale and computational features	Focus	Typical applications in digital twin
Finite element analysis	Continuum mechanics	Structural scale, high computing cost (hours-days)	Stress, strain, deformation	Aids and appliances - organizational interface stress assessment Structural fatigue life prediction
Multibody dynamics simulation	Rigid/Flexible dynamics	System scale, Computational efficiency (seconds-minutes)	Joint moment, motion trajectory	Simulation of assistive device system dynamics behavior, quantitative assessment of assistive effects
Musculoskeletal simulation	Multibody dynamics and muscle physiology	Musculoskeletal system scale, Compute medium (minutes-hours)	Muscle strength, activation level	Evaluation of neuromuscular control strategies, analysis of assistive biomechanical mechanisms

With the incorporation of real-time sensing data, digital twin models can more accurately reflect patient-specific physiological conditions and movement patterns across different temporal scales and usage scenarios (Uhlenberg et al., 2023). Consequently, DT systems evolve from conventional performance-simulation tools into dynamically updated predictive mechanisms.

For example, Derungs et al. (Derungs and Amft, 2020) demonstrated that simulations based on personalized biomechanical models can identify sensor locations that maximize sensitivity to specific gait abnormalities or muscle activation patterns. Their findings showed that the optimal sensor configuration changes as a patient’s functional abilities recover over time, providing important support for the development of intelligent interactive DT systems capable of adapting to evolving user conditions.

Through such data-assimilation mechanisms, DT systems can interpret changes in gait stability, muscle recovery trends, and the evolution of local structural loading within assistive devices. As a result, digital twins gain the capability for continuous monitoring, state assessment, and early risk warning, thereby enabling more proactive and personalized rehabilitation interventions.

### 3.4 Chapter summary

This chapter proposed a lifecycle-oriented digital twin framework for rehabilitation assistive devices, encompassing multimodal data acquisition, digital modeling, simulation and optimization, manufacturing integration, and closed-loop feedback updating. The framework provides a unified conceptual architecture capable of supporting diverse categories of rehabilitation assistive devices while accommodating their distinct functional requirements.

More importantly, the chapter highlights that the effectiveness of DT technologies in rehabilitation engineering depends not only on individual enabling technologies but also on the coordinated integration of sensing, modeling, simulation, manufacturing, and feedback processes throughout the entire device lifecycle. Such lifecycle-wide collaboration forms the foundation for achieving personalized design, adaptive optimization, and intelligent decision-making in future rehabilitation assistive devices.

## 4 Digital-twin-driven intelligent manufacturing and production closed loop

In a digital-twin-based rehabilitation assistive device system, the manufacturing stage serves as the critical link that transforms virtual models into physical entities and validates their performance (Liu et al., 2024). The optimal design solutions generated in the virtual environment—based on multimodal modeling, performance simulation, and optimization—must be accurately materialized through manufacturing (Ogasawara et al., 2024). Meanwhile, process data generated during fabrication feed back into the virtual model, enabling continuous updates and establishing a complete cyber–physical collaborative chain. Traditional assistive device manufacturing relies on static design and manual adjustment, making it difficult to satisfy the needs for individualized fitting and dynamic optimization. The introduction of digital twins transforms manufacturing from a linear and passive execution process into a dynamic production closed loop equipped with real-time sensing, predictive inference, and adaptive control capabilities. Its core lies in the deep integration of data flow, model flow, and control flow, thereby enhancing manufacturing accuracy, process stability, and product consistency through cyber–physical cooperation. This chapter systematically elaborates the intelligent manufacturing logic and production closed loop of rehabilitation assistive devices under digital-twin guidance from three perspectives: manufacturing system architecture, the twin-enabled applications of additive manufacturing, and data-driven feedback mechanisms.

### 4.1 Overall architecture of the digital twin manufacturing system

The digital twin manufacturing system aims to achieve high-fidelity mapping and bidirectional collaboration between virtual design, process planning, and physical manufacturing, thereby constructing a production closed loop that is perceptive, predictable, and optimizable, as illustrated in Figure 6.

**FIGURE 6:**
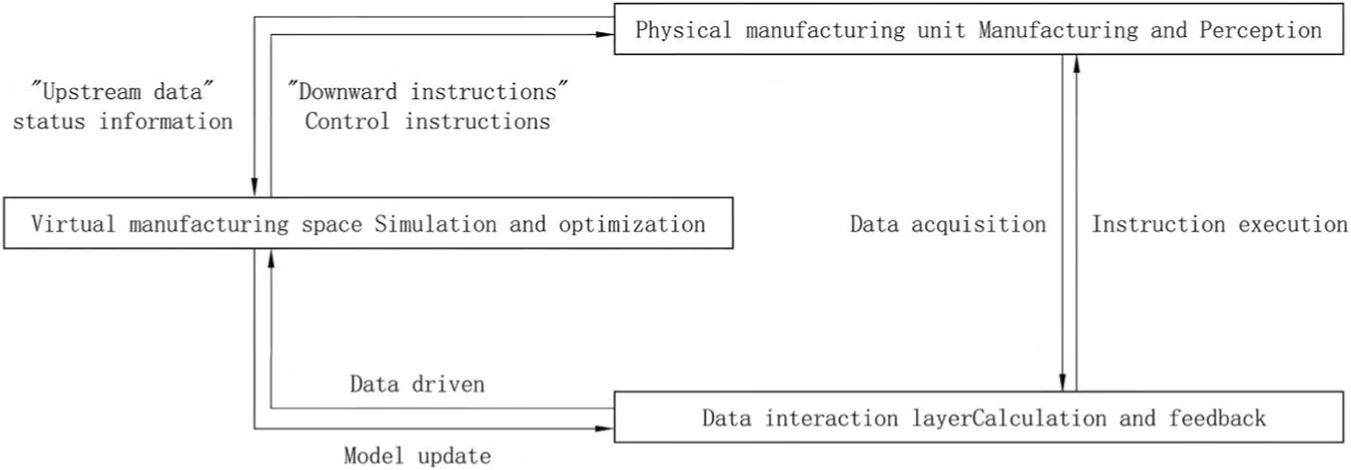
Core framework diagram of the digital twin manufacturing system.

In this system, virtual manufacturing performs the core functions of process modeling and workflow simulation. Through finite element analysis, thermodynamic simulation, and topology optimization, it generates individualized manufacturing parameters and toolpath planning for assistive devices. For example, Ali et al. (Ali et al., 2021) developed a carbon-fiber-reinforced polymer 3D-printed ankle–foot orthosis using finite element analysis and multi-material optimization. Their design reduced equivalent stress at key regions by approximately 31% while maintaining joint flexibility, significantly improving mechanical performance and individual fit.

Physical manufacturing consists of additive manufacturing equipment, environmental monitoring systems, and execution control modules. It is responsible for transforming virtual designs into physical products while continuously producing high-frequency process data such as melt-pool temperature, interlayer bonding quality, and material solidification behavior. Stavropoulos et al. (Stavropoulos et al., 2021) employed a robust control method based on linear matrix inequalities to address uncertainties in material properties affecting melt-pool thermal stability. Their approach achieved a 68% faster response time than conventional PID controllers, greatly enhancing robustness in additive manufacturing.

The data interaction layer bridges the virtual and physical spaces by enabling real-time acquisition, synchronization, and transmission of multidimensional data through communication protocols and edge computing frameworks. This allows the virtual model to continuously update structural states and process parameters during manufacturing. For instance, Pantelidakis et al. (Pantelidakis et al., 2022) integrated the Unity real-time development platform with multi-source data acquisition to create a digital twin ecosystem for FDM additive manufacturing. Their system achieved near–real-time synchronization and bidirectional communication for position, temperature, and system status, offering a low-cost and reliable pathway for upgrading traditional manufacturing equipment.

The core of this architecture lies in constructing a bidirectional feedback production closed loop:Top-down: the virtual model drives physical manufacturing through inference and prediction, enabling online adjustment of toolpaths, energy input, and process parameters; Bottom-up: sensor data continuously calibrate the virtual model, allowing updates to material properties, boundary conditions, and process precision to improve cyber–physical consistency. Jyeniskhan et al. (Jyeniskhan et al., 2023) integrated the EfficientDet-Lite machine learning model with a Unity-based digital twin platform to establish a bidirectional communication and real-time monitoring system for FDM printing. Their method achieved an average defect detection accuracy of 92%, significantly improving printing reliability and product quality.

### 4.2 Applications of additive manufacturing in digital twin systems

Additive manufacturing (AM), due to its high geometric freedom, strong customizability, and programmable process parameters, is one of the manufacturing methods most compatible with digital-twin methodologies for rehabilitation assistive devices (Rafiq et al., 2025). Digital twins directly map simulation-optimized structural features, functional parameters, and material layouts onto the AM workflow, enabling rapid fabrication of individualized structures.

In the virtual modeling stage, the system automatically generates optimal internal structures and material gradients based on simulated stress distribution, fatigue life, and deformation characteristics. During fabrication, AM equipment carries out layer-by-layer deposition following digital-twin-derived parameters while embedded sensors monitor thermal fields, melt-pool stability, and forming quality. Chen et al. (Chen et al., 2025) proposed a digital-twin-based real-time decision-making framework combining time-series deep neural networks (TiDE) and model predictive control (MPC). Their approach enabled multi-step prediction of melt-pool temperature and depth, dynamically regulating laser power to suppress porosity defects while satisfying dilution constraints—significantly enhancing the forming quality and closed-loop control of directed energy deposition. In assistive device manufacturing, such methods have been validated in ankle–foot orthosis shells, exoskeleton joint housings, and flexible support components. For example, Banga et al. (Banga et al., 2018) coupled finite element analysis with additive manufacturing to perform multi-scheme mechanical simulations for ankle–foot orthosis design. They ultimately selected a configuration whose mechanical behavior closely matched real products while ensuring structural rigidity and ventilation performance, thus improving comfort and functional adaptability.

Through cyber–physical collaboration, AM not only supports structural fabrication but also acts as a crucial feedback loop for validating simulation outcomes and refining design parameters. This enables true full life-cycle closed-loop integration between design and manufacturing.

### 4.3 Data-feedback-driven manufacturing optimization and quality control

In the digital twin manufacturing system, the multimodal data streams generated during fabrication constitute the driving force of the closed-loop design process, enabling dynamic adjustment and long-term evolution of manufacturing precision, material quality, and process stability. Zhou et al. (Zhou et al., 2020) constructed a knowledge-driven digital twin manufacturing unit integrating dynamic knowledge bases and intelligent knowledge-guided skills. Their system achieved self-perception, self-decision-making, and self-optimization in the manufacturing process, addressing the limitations of traditional systems that rely heavily on manual intervention and lack autonomous learning. Demonstrations in machining scenarios verified the feasibility of intelligent process planning, dynamic scheduling, and process control. This data-driven optimization ultimately forms a production closed loop composed of predictive models, feedback pathways, and decision algorithms. As a result, rehabilitation assistive device manufacturing can continuously enhance precision, optimize process parameters, and reduce quality fluctuations as data accumulate.

Machine learning–based and data-driven models also play a growing role in the production closed loop. In rehabilitation assistive device manufacturing, such methods have been used to automatically regulate deposition density, control geometric deviations, and update virtual model parameters using process data. This allows the digital twin to synchronize updates of material properties, structural constraints, and manufacturing errors, thereby improving its predictive accuracy and adaptability. Khan et al. (Khan et al., 2025) integrated digital twins with machine learning algorithms to monitor and predict forming defects and performance deviations in polymer additive manufacturing in real time. Their method enabled dynamic optimization and closed-loop control of process parameters, significantly improving manufacturing consistency and product reliability.

### 4.4 Comparative analysis of digital twin and traditional methods

The integration of digital twin technology not only transforms the design and manufacturing workflow of rehabilitation devices but also provides significant advantages across multiple dimensions compared to traditional approaches. Table 3 presents a systematic comparison across key aspects—including design efficiency, individualized adaptation, system adaptability, feedback mechanisms, manufacturing flexibility, personalization implementation, user acceptance, and cost-effectiveness—while incorporating quantitative evidence from existing studies to illustrate the specific contributions of digital twin technology to enhancing rehabilitation device performance.

**TABLE 3:** Comparison of digital twin and traditional rehabilitation aids design methods.

Dimension	Traditional methods	Digital twin method	Core differences
Design cycle	Learn from trial and error, from weeks to months	Virtual simulation, from a few days to a few weeks	Iteration accelerates, and virtual replaces physical objects
Personalization accuracy	Reliant on statistical models, limited adaptation	Data fusion modeling, precise individual adaptation	Improved accuracy and optimized biomechanical adaptation
System adaptability	Static design, difficult to adjust	Real-time data driven, dynamic updates	From static to continuous adaptation
Feedback mechanism	Follow-up feedback, high delay	Sensor real-time closed-loop feedback	Real-time response, data-driven optimization
Manufacturing flexibility	Mold standard process, high modification cost	Additive manufacturing, rapid personalized production	Flexible manufacturing, small batches are feasible
Personalized paths	Morphological fit, fixed function	Dynamic functional symbiosis, evolving with rehabilitation	From lamination to symbiosis, multi-dimensional adaptation
User acceptance	Dependent on subjective perception of the finished product	Virtual trial, quantified effectiveness validation	Trust moves forward and the effect is visualized
Cost-effectiveness	Focus on unit manufacturing costs	Total life cycle cost, rehabilitation benefits	The total cost of the system is better

In summary, digital twin technology systematically surpasses the limitations of traditional rehabilitation device development across multiple dimensions. Its core advantage lies in establishing a full-chain closed loop from user data to virtual models, physical devices, and real-time feedback, transforming rehabilitation devices from static products into dynamic intelligent systems. Although challenges remain in data standardization, real-time modeling, and system integration, digital twin technology provides a critical technical pathway and empirical foundation for advancing rehabilitation engineering toward precision, personalization, and intelligence.

## 5 Applications, clinical translation, and future perspectives of digital twins in rehabilitation assistive devices

### 5.1 Representative applications across different categories of rehabilitation assistive devices

Digital twin technologies have been increasingly applied across a wide range of rehabilitation assistive devices. However, their implementation pathways vary according to device functionality and the complexity of human–device interactions.

For structure-oriented rehabilitation devices, such as ankle–foot orthoses, spinal orthoses, and passive prosthetic components, DT technologies are primarily employed for personalized design, biomechanical simulation, structural optimization, and additive-manufacturing-enabled customization. By integrating medical imaging, finite element analysis, and patient-specific manufacturing workflows, DT systems can substantially improve device fit, comfort, and mechanical performance. For example, Lara-Velazquez et al. (Lara-Velazquez et al., 2024) jointly optimized control parameters and mechanical characteristics of AFOs, achieving accurate ankle-angle tracking while significantly reducing system oscillations and energy consumption. Similarly, Zhang et al. (Zhang et al., 2025) proposed a multi-objective human-in-the-loop optimization framework for simulated drop-foot gait, balancing improvements in foot clearance and gait symmetry. Their approach achieved simultaneous enhancements in lower-limb kinematics and overall gait symmetry.

For human–device coupled rehabilitation devices, including adaptive prostheses and intelligent orthotic systems, DT technologies enable continuous monitoring of biomechanical performance and user adaptation. Through wearable sensing technologies and musculoskeletal modeling, virtual models can dynamically reflect changes in gait patterns, loading conditions, and rehabilitation progress. PMücke et al. (Mücke et al., 2024) developed a low-dimensional surrogate model based on a Wasserstein autoencoder and Transformer architecture, enabling real-time data assimilation and uncertainty quantification while maintaining high prediction accuracy. This approach substantially improved state and parameter estimation efficiency for nonlinear intelligent orthotic systems. Hussain et al. (Hussain et al., 2025) performed time-dependent reliability analysis of underactuated prosthetic devices using surrogate modeling and feedforward neural networks to predict global dynamic responses, thereby enhancing reliability assessment under time-varying uncertainties.

For intelligent interactive rehabilitation systems, such as powered exoskeletons and rehabilitation robots, DT technologies increasingly support predictive control, adaptive decision-making, and human–machine interaction optimization. These systems require continuous synchronization between physical devices and virtual environments and represent the most advanced form of rehabilitation DT implementation. Zhang et al. (Zhang et al., 2022) developed a hybrid artificial neural network integrating neuromusculoskeletal physics-based models with transfer-learning techniques to improve cross-subject ankle-torque prediction. Their approach significantly enhanced the generalization capability of personalized exoskeleton control under limited training data. Rose et al. (Rose et al., 2022) proposed a model-free deep reinforcement learning framework based on Deep Deterministic Policy Gradient (DDPG) for end-to-end control of hip–knee–ankle exoskeletons. Trained and evaluated within a three-dimensional OpenSim-RL environment, the controller achieved mean absolute errors below 0.67° across all joints and demonstrated robust tracking performance for unseen gait patterns and diverse user conditions.

Collectively, these examples indicate that although implementation strategies vary among device categories, successful DT systems consistently rely on the integration of sensing, modeling, simulation, manufacturing, and feedback mechanisms within a unified lifecycle-oriented framework, Table 4.

**TABLE 4:** Representative applications of digital twins in rehabilitation assistive devices.

Device type	DT functione	Key technology	Validation method	Main outcome
Structure-oriented-rehabilitation devices	Design optimization	FEA + AM	Clinical trial	Improved rehabilitation efficiency
Human-device coupled rehabilitation devices	Gait prediction	AI + AM	Gait analysis	Enhanced adaptation
Intelligent interactive rehabilitation devices	Adaptive control	AI + DT	User fit evaluation	Improved comfort

### 5.2 Clinical translation challenges and user-centered validation strategies

Despite substantial advances in rehabilitation DT technologies, their ultimate success depends on clinical effectiveness and user acceptance (Rafiq et al., 2025). Consequently, clinical translation should not be regarded as a post-development activity but rather as an integral component of the DT lifecycle.

User-Centered Design (UCD) principles emphasize the active involvement of patients, clinicians, caregivers, and rehabilitation specialists throughout the development process. Such approaches can significantly improve device usability, comfort, accessibility, and long-term adherence. International standards, including ISO 9241-210 (ergonomics of human–system interaction), ISO 13485 (quality management systems for medical devices), and ISO 14971 (risk management for medical devices), provide important guidance for rehabilitation-device development from human-factors, quality-management, and risk-control perspectives.

Importantly, validation of rehabilitation DT systems should extend beyond technical performance metrics. A comprehensive evaluation framework should incorporate patient-reported outcomes, clinician assessments, biomechanical indicators, functional rehabilitation measures, and safety-related parameters. Only through rigorous multidimensional validation can DT technologies progress from laboratory demonstrations to widespread clinical deployment.

Nevertheless, several critical challenges continue to hinder large-scale implementation. From a technical perspective, one of the primary obstacles is the acquisition and integration of heterogeneous multimodal data. Rehabilitation DT systems typically rely on wearable sensors, medical imaging, biomechanical measurements, manufacturing records, and clinical evaluations. Ensuring data consistency, temporal synchronization, and interoperability across these sources remains a significant challenge. Furthermore, the development of personalized DT models requires accurate subject-specific parameters, while substantial inter-individual variability in anatomy, biomechanics, and rehabilitation responses limits model generalizability and scalability. Computational efficiency also remains a major concern. Although DT systems are often envisioned as real-time decision-support platforms, high-fidelity simulations impose substantial computational demands. Reduced-order models and machine-learning-based surrogate models offer promising solutions, yet balancing computational efficiency and predictive accuracy remains an active area of research.

Clinical translation presents additional challenges. Rehabilitation outcomes depend not only on device performance but also on patient adherence, integration into clinical workflows, and long-term user engagement. Consequently, DT validation must extend beyond engineering metrics to include patient-reported outcomes, clinician evaluations, usability assessments, and functional rehabilitation indicators. However, standardized validation protocols for rehabilitation DT systems remain underdeveloped.

Regulatory and standardization issues also warrant greater attention. As rehabilitation DT technologies evolve into clinical decision-support systems and personalized medical devices, compliance with relevant regulatory frameworks becomes increasingly important. Applicable standards include ISO 13485 (quality management), ISO 14971 (risk management), ISO 10993 (biocompatibility), and the ASTM F42 series for additive manufacturing. Regulatory pathways established by the U.S. Food and Drug Administration (FDA) and the European Medical Device Regulation (MDR) are expected to play a pivotal role in shaping future clinical deployment.

Overall, overcoming these technological, clinical, and regulatory challenges will require close collaboration among engineers, clinicians, rehabilitation specialists, manufacturers, and regulatory agencies. Such interdisciplinary cooperation is essential for translating rehabilitation DT technologies from conceptual frameworks into clinically impactful solutions.

### 5.3 Future trends and research opportunities

The convergence of DT technologies with artificial intelligence, advanced sensing technologies, and intelligent manufacturing is expected to fundamentally reshape the future of rehabilitation engineering. Several emerging directions are likely to drive the next-generation of rehabilitation DT systems.

One prominent trend is the integration of artificial intelligence and foundation models into DT platforms. Recent advances in large-scale machine learning have demonstrated unprecedented capabilities in knowledge representation, multimodal reasoning, and predictive analytics. Future rehabilitation DT systems may leverage these capabilities to support intelligent rehabilitation planning, automated model updating, personalized intervention strategies, and adaptive decision-making.

Another promising direction involves the development of digital human technologies. By integrating anatomical, biomechanical, physiological, and behavioral information into unified virtual representations, digital humans can provide a more comprehensive description of individualized rehabilitation processes. Such developments are expected to substantially enhance personalization and predictive performance in rehabilitation DT systems.

Cloud–edge collaborative architectures are also expected to become increasingly important. Edge computing enables low-latency data processing and local decision-making, whereas cloud platforms provide scalable storage, model training, and cross-institutional collaboration capabilities. The integration of cloud and edge infrastructures will facilitate large-scale deployment of DT systems across hospitals, rehabilitation centers, and home-based rehabilitation environments.

Furthermore, future DT systems are expected to evolve from passive monitoring platforms into adaptive and self-evolving intelligent systems. Through continuous interaction with users, clinicians, and rehabilitation environments, DT systems will progressively improve their predictive accuracy and decision-making capabilities.

Finally, future research should place greater emphasis on interdisciplinary integration, clinical translation, and standardized evaluation methodologies. Establishing open-data platforms, interoperable DT ecosystems, and shared validation frameworks will be critical for accelerating technology adoption and improving rehabilitation outcomes.

## 6 Conclusion

Digital twin technology is emerging as a key enabler for transforming the design, development, manufacturing, and deployment of rehabilitation assistive devices. By establishing continuously synchronized, bidirectional, and dynamically evolving connections between physical and virtual systems, DT technology enables accurate performance prediction, optimization, personalized design, intelligent control, and lifecycle management. With the rapid advancement of rehabilitation engineering, artificial intelligence, advanced manufacturing, and digital healthcare technologies, DT is evolving from a digital representation tool into a core infrastructure for next-generation intelligent rehabilitation systems.

This review systematically summarized the current state of research and applications of DT technologies in rehabilitation assistive devices. To address the lack of a unified analytical perspective in existing studies, a DT-oriented taxonomy was established, categorizing rehabilitation assistive devices into structure-oriented devices, human–device coupled devices, and intelligent interactive devices. The distinct DT requirements, data characteristics, and system complexities associated with each category were analyzed. Building upon this taxonomy, a lifecycle-oriented DT framework was proposed, integrating data acquisition, digital modeling, simulation and optimization, manufacturing integration, and closed-loop feedback updating into a unified architecture. This framework provides a comprehensive perspective for understanding the implementation pathways and operational mechanisms of DT systems in rehabilitation engineering.

The review further highlights that the development of high-fidelity and real-time rehabilitation DT systems relies on the synergistic integration of biomechanical modeling, finite element analysis, multibody dynamic simulation, artificial intelligence, multi-source data fusion, and additive manufacturing technologies. Different categories of rehabilitation assistive devices exhibit distinct implementation priorities: structure-oriented devices emphasize personalized design and structural optimization; human–device coupled devices focus on biomechanical adaptation and user-specific interaction; whereas intelligent interactive devices require advanced sensing, adaptive control, and intelligent decision-making capabilities. Existing evidence suggests that DT technologies can significantly improve design efficiency, performance prediction, adaptability, and rehabilitation service quality, demonstrating substantial potential for precision rehabilitation and personalized healthcare.

Despite these advances, several challenges remain to be addressed. These include the integration and interoperability of heterogeneous multimodal data, real-time computation of complex biomechanical models, establishment of lifecycle-wide closed-loop updating mechanisms, development of standardized clinical validation protocols, and compliance with regulatory and certification requirements. Addressing these challenges will require close collaboration among rehabilitation medicine, mechanical engineering, artificial intelligence, materials science, and regulatory communities.

Looking forward, DT technologies are expected to become increasingly integrated with artificial intelligence, digital human technologies, cloud–edge computing, advanced sensing systems, and intelligent manufacturing. Future rehabilitation DT systems will likely evolve from passive monitoring platforms into adaptive and self-evolving intelligent systems capable of continuous learning, autonomous optimization, and predictive decision-making. Such developments have the potential to enable real-time coordination and intelligent optimization throughout the entire rehabilitation lifecycle, spanning design, manufacturing, deployment, and clinical evaluation. Ultimately, DT technology is expected to serve as a foundational platform for intelligent rehabilitation ecosystems connecting patients, clinicians, engineers, and manufacturers, thereby supporting the advancement of precision rehabilitation and smart healthcare.

In summary, DT technology not only serves as a bridge between physical and digital worlds but also represents a promising platform for the development of next-generation rehabilitation assistive devices. Through continued technological innovation, interdisciplinary collaboration, and rigorous clinical validation, DT technologies are expected to play an increasingly important role in improving rehabilitation outcomes, optimizing healthcare resources, and enabling personalized rehabilitation services.
