# Supplementary material for: Research progress on digital-twin-driven full life-cycle design and manufacturing of rehabilitation assistive devices
Source: Front Bioeng Biotechnol. 2026 Sep 15;14:1820883. doi: 10.3389/fbioe.2026.1820883 (PMC13620225; doi:10.3389/fbioe.2026.1820883)
Supplement: Supplementary file 1 [file Supplementaryfile1.docx]

Research Progress on Digital-Twin-Driven Full Life-Cycle Design and Manufacturing of Rehabilitation Assistive Devices

# 1 Supplementary Figures and Tables

## 1.1 Supplementary Figures

Figure1 Schematic diagram of structure-oriented rehabilitation equipment

Figure2 Schematic diagram of human-machine collaborative rehabilitation equipment

Figure3 Schematic diagram of intelligent interactive rehabilitation equipment

Figure4 Digital twin modeling workflow for rehabilitaion assistive devices

Figure5 Digital Twin Mapping Framework

Figure6 Core Framework Diagram of the Digital Twin Manufacturing System

## 1.2 Supplementary Tables

Table1 Digital-Twin-Oriented Taxonomy and Functional Requirements of Rehabilitation Assistive Devices

| **Category** | **Representative Devices** | **Primary DT Objective** | **Data Requirement** | **Real-Time Requirement** | **Manufacturing Integration** |
| --- | --- | --- | --- | --- | --- |
| Structure-oriented | AFO, KAFO, Passive Prosthesis... | Structural optimization | Geometry,Mechanics | Low | High |
| Human-device coupled | Prosthesis, Smart Orthosis... | Biomechanical adaptation | Gait,Interaction | Medium | Medium |
| Intelligent interactive | Exoskeleton, Rehabilitation Robot... | Control and prediction | Multimodal SensorData | High | Medium |

Table2 Core simulation strategies in digital twin

| **Simulation strategy** | **Core principle** | **Scale and computational features** | **Focus** | **Typical applications in digital twin** |
| --- | --- | --- | --- | --- |
| Finite element analysis | Continuum Mechanics | Structural scale, high computing cost (hours-days) | Stress, strain, deformation | Aids and Appliances - Organizational Interface Stress Assessment,  Structural Fatigue Life Prediction |
| Multibody dynamics simulation | Rigid/Flexible dynamics | System scale,Computational efficiency (seconds-minutes) | Joint moment, motion trajectory | Simulation of assistive device system dynamics behavior, quantitative assessment of assistive effects |
| Musculoskeletal simulation | Multibody dynamics and muscle physiology | Musculoskeletal system scale,Compute Medium (minutes-hours) | Muscle strength, activation level | Evaluation of neuromuscular control strategies, analysis of assistive biomechanical mechanisms |

Table3 Comparison of Digital Twin and Traditional Rehabilitation Aids Design Methods

| **Dimension** | **Traditional methods** | **Digital twin method** | **Core differences** |
| --- | --- | --- | --- |
| Design cycle | Learn from trial and error, from weeks to months | Virtual simulation, from a few days to a few weeks | Iteration accelerates, and virtual replaces physical objects |
| Personalization accuracy | Reliant on statistical models, limited adaptation | Data fusion modeling, precise individual adaptation | Improved accuracy and optimized biomechanical adaptation |
| System adaptability | Static design, difficult to adjust | Real-time data driven, dynamic updates | From static to continuous adaptation |
| Feedback mechanism | Follow-up feedback, high delay | Sensor real-time closed-loop feedback | Real-time response, data-driven optimization |
| Manufacturing flexibility | Mold standard process, high modification cost | Additive manufacturing, rapid personalized production | Flexible manufacturing, small batches are feasible |
| Personalized paths | Morphological fit, fixed function | Dynamic functional symbiosis, evolving with rehabilitation | From lamination to symbiosis, multi-dimensional adaptation |
| User acceptance | Dependent on subjective perception of the finished product | Virtual trial, quantified effectiveness validation | Trust moves forward and the effect is visualized |
| Cost-effectiveness | Focus on unit manufacturing costs | Total Life Cycle Cost, Rehabilitation Benefits | The total cost of the system is better |

Table4 Representative Applications of Digital Twins in Rehabilitation Assistive Devices

| **Device Type** | **DT Functione** | **Key Technology** | **Validation Method** | **Main Outcome** |
| --- | --- | --- | --- | --- |
| Structure-Oriented-Rehabilitation Devices | Design Optimization | FEA+AM | Clinical Trial | Improved Rehabilitation Efficiency |
| Human-Device Coupled Rehabilitation Devices | Gait Prediction | AI+AM | Gait Analysis | Enhanced Adaptation |
| Intelligent Interactive Rehabilitation Devices | Adaptive Control | AI + DT | User Fit Evaluation | Improved Comfort |
